# Deciduosis in a cesarean scar

**DOI:** 10.4322/acr.2021.383

**Published:** 2022-05-06

**Authors:** Toyaja Jadhav, Rohini Doshetty

**Affiliations:** 1 12 Airforce Hospital, Department of Laboratory Sciences, Gorakhpur, Uttar Pradesh, India

**Keywords:** Cesarean Section, Embryo implantation, Cicatrix

## Abstract

Deciduosis is the presence of ectopic decidual tissue outside the uterus, pelvic, or abdominal organs usually associated with pregnancy. Cutaneous deciduosis is a highly uncommon manifestation of deciduosis and most commonly is misdiagnosed as a primary malignancy or a metastatic deposit. Typically, it is detected incidentally during operative procedures. It has been rarely documented within a surgical scar; with the incidence of surgically proven deciduosis being approximately 1.6%, and is often difficult to diagnose due to its rarity. Here, we present a case of deciduosis of cesarean scar in a 34-year-old pregnant female.

## INTRODUCTION

Deciduosis is clinically defined as the presence of ectopic decidual tissue in locations outside the uterus. It has been reported to occur in various pelvic and extra-pelvic sites. It is typically known to present during pregnancy but has also been reported in non-pregnant women. It is considered to be a benign lesion during pregnancy, not associated with any obstetric complications. It does not have any pathological impact on the mother as well as the fetus. It is usually asymptomatic and can remain undetected throughout pregnancy. Total remission is generally known to occur in the postpartum period; however, some cases may require surgical intervention, especially those that often mimic a malignancy.^1^

Cutaneous deciduosis is an uncommon manifestation of cutaneous endometriosis. Cutaneous endometriosis usually occurs within the umbilical region or in abdominal surgical scars, the latter typically occurring after cesarean section, appendectomy, or an inguinal hernia repair. Although deciduosis has been reported in numerous ectopic locations, most lesions are encountered within the cervix or ovary.^2^

To date, only a few well-documented instances of cutaneous deciduosis have been reported, with very few occurring within abdominal scars from previous cesarean sections.^2^^,^^3^

We present a case of cutaneous deciduosis of a cesarean scar diagnosed incidentally in a 34-year-old pregnant female, along with some review of the available literature.

## METHODOLOGY

All the case series and case reports, inclusive of their references, identified by extensively searching the PubMed, Scopus, Medline and Google Scholar databases utilizing the keywords “deciduosis”, “extra- abdominal deciduosis”, “cutaneous deciduosis” and “deciduosis in a cesarean scar” were read and included in this manuscript. A total of 13 cases of cutaneous deciduosis have been reported in literature from 1982; of these, 8 of them have been known to occur in a scar of a previous cesarean section.

## CASE REPORT

A 34-year-old pregnant female (G2P1L1A0) presented to this hospital at 38 weeks of gestation with complaints of abdominal pain.

Her previous pregnancy was six years ago, which had concluded in a healthy child with breech presentation, delivered through lower segment cesarean section (LSCS). She was a known case of hypothyroidism and was also suffering from Gestational Diabetes Mellitus (GDM) in the current pregnancy and was being managed for the same with Tab Eltroxin 50ug, Inj Glargine, and Tab Metformin 500mg, respectively.

Her obstetric examination revealed a uterine fundus height of 36 weeks with a breech presentation and a normal fetal heart rate (FHR). Her preliminary hematological as well as serological investigations were within normal clinical limits.

Obstetric ultrasound examination was also carried out, which revealed an adequate Amniotic Fluid Index (AFI) with the placenta placed anteriorly and a breech fetal presentation.

Hence, in view of the above clinical and ultrasonographical findings, the patient was taken for elective LSCS as a case of Antenatal Case (ANC) with breech presentation with previous LSCS with Gestational DM and hypothyroidism.

A healthy infant was delivered. Additionally, intraoperatively, the scar of previous LSCS presented with features of endometriosis along the left lateral margin, which was excised clinically as endometrioma and sent for histopathological evaluation.

Grossly, the sample presented as multiple fragmented tissue bits, with the largest measuring approximately 2cm and the smallest measuring approximately 1cm in its greatest dimension, respectively.

**Figure 1:**
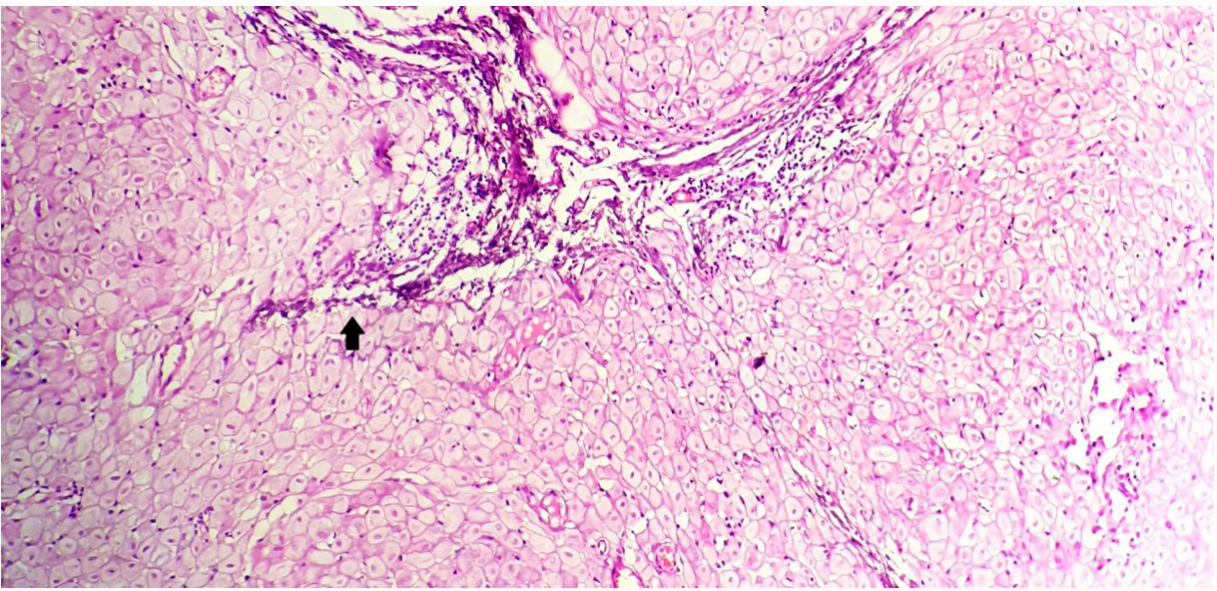
Photomicrograph of the lesion reveals multiple nodules composed of decidualized stromal cells surrounding a few slit-like endometrial glands admixed with fibroblasts and collagen. The arrow highlights the slit-like endometrial glands (H&E, 40x).

On microscopic evaluation, hematoxylin and eosin (H&E) stained sections revealed multiple nodules composed of decidualized stromal cells surrounding a few slit-like endometrial glands with fibroblasts and collagen (Figure 1).

**Figure 2:**
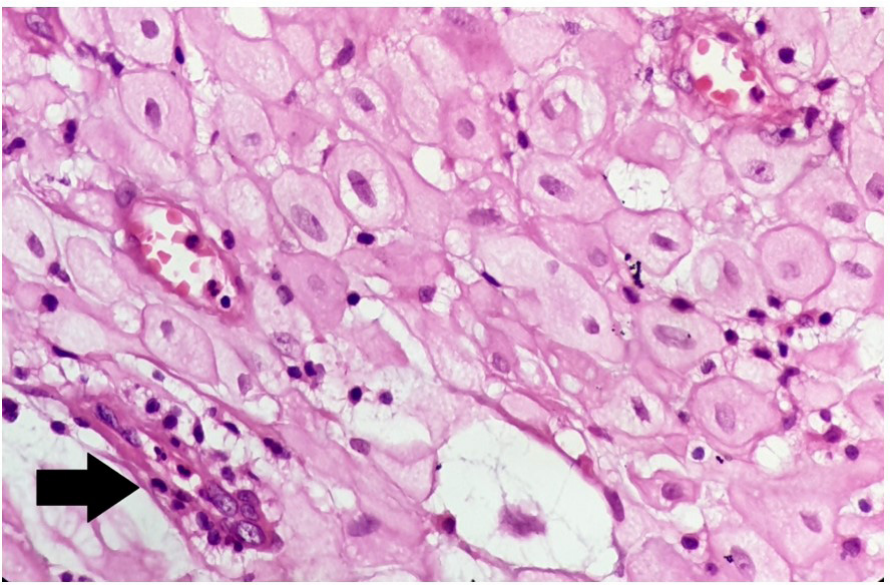
Photomicrograph of the lesion. High power view of the lesion showing the round to polygonal structure of decidual cells. The arrow highlights slit-like endometrial glands (H&E;100x).

These decidual cells were polygonal, with large nuclei, abundant homogenous eosinophilic cytoplasm (Figure 2), and associated with vacuolar degeneration in some places.

**Figure 3:**
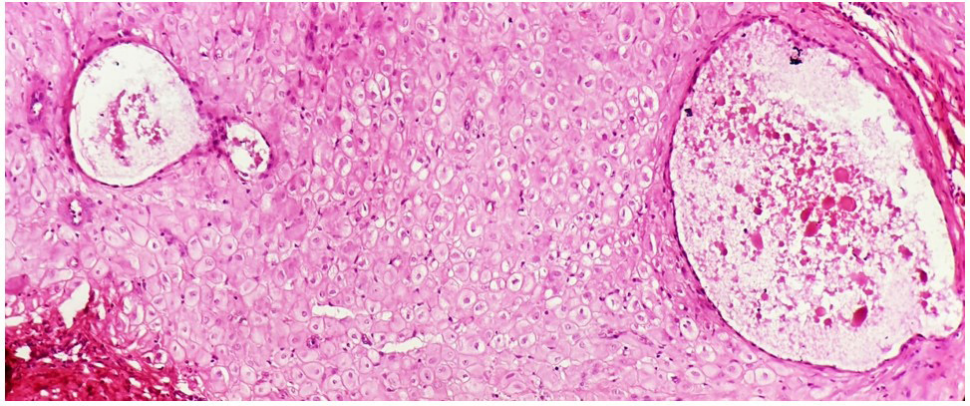
Photomicrograph of occasional dilated endometrial glands containing eosinophilic secretions seen lying amongst the decidual cells (H&E, 40x).

Occasional dilated endometrial glands were also noted, which showed eosinophilic secretions with adipose tissue present along the periphery (Figure 3). No features depicting atypia were noted.

**Figure 4:**
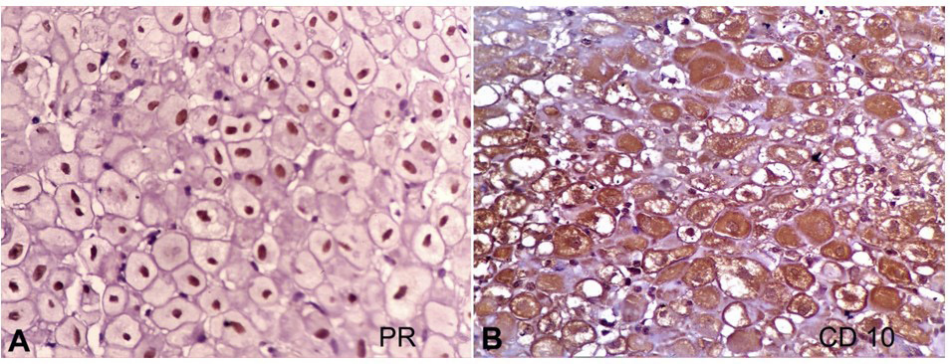
**A** and **B –** decidual cells showing positive reaction for PR and CD10 respectively (400x).

On immunohistochemical evaluation, it was noted that the decidual cells showed reactivity to PR receptors and CD10 antibody (Figure 4).

These histopathological features, along with the clinical findings connoted with the diagnosis of deciduosis of cesarean scar.

## DISCUSSION

Deciduosis is clinically defined as the presence of decidual tissue at sites other than the uterus.^1^ Walker^4^ first described it in 1887. Extrauterine decidual cell deposition is most commonly seen in the ovaries, cervix, uterine serosa, and the lamina propria of the fallopian tubes, while it is less commonly noted along the appendix, omentum, diaphragm, liver, spleen, paraaortic-pelvic lymph nodes and renal pelvis. Involvement of a previous surgical scar is uncommon. The incidence of a surgically proven cutaneous deciduosis is approximately 1.6%.^3^ It is commonly associated with pregnancy, as seen in our case. However, it can also be seen in a non-pregnant state. It is associated with a progesterone-secreting active corpus luteum or the adrenal cortex in the non-pregnant condition. Most patients are asymptomatic. However, some may present with features of hematuria or even obstructed ileus due to the involvement of various organs.^5^ Our patient was asymptomatic.

Grossly, deciduosis of cesarean scar may show a varied presentation ranging from an individual geographic pattern, nodular distribution or a polypoid appearance, which may often mimic a neoplasm.

Microscopically, decidual cells are commonly found to be associated with endometrial tissue present over a scar. The lesions may frequently present as small cell groups or single-cell clusters, and uncommonly, they are in the form of widespread-diffuse deciduosis that completely occupies the scar along with the underlying adipose stroma. Our case presented with microscopic features depicting the latter. Decidual cells are generally large and polygonal, with homogeneous, eosinophilic cytoplasm associated with varying degrees of vacuolar degeneration. Decidual cell vacuolization is related to the duration of the pregnancy. Stroma may also show myxoid deposit due to vacuole rupture if the decidual cell cytoplasmic vacuolar degeneration is over 50%.^5^ Our case did present some degree of vacuolar degeneration; however, myxoid changes were not noted.

The pathogenesis of ectopic decidual reactions is not yet fully understood. It is still not completely deciphered whether it is a physiological reaction or a pathological process. It is said to be the result of the exaggerated response of the endometrium to progesterone during pregnancy.^5^

Zaytsev and Taxy^6^ have suggested two related theories. The most commonly accepted theory is the metaplasia of the sub-celomic pluripotent mesenchymal cells with the effect of progesterone. The fact that the lesion resolves once the hormonal stimulus ends supports this theory. Another theory is the “de novo” development of decidual cells. Endometriotic foci undergo marked stromal decidualization with the effect of progesterone during pregnancy, which resembles ectopic decidua. It is, therefore, necessary to differentiate deciduosis from decidualized endometriosis clinicopathologically. The presence of clinical symptoms at the beginning of the menstrual period and the presence of endometriotic foci in other areas is important for cases with a clinical picture of endometriosis.^5^ Our patient did not present with any clinical features or a history confirming previous endometriosis. Histologically, the diffuse distribution of the lesion, edema of the decidualized stroma, old and new hemorrhagic foci, presence of pseudoxanthoma cells and fibrosis signifying endometrial gland atrophy and Arias-Stella reaction are important diagnostic features of decidual transformation of endometriotic foci in pregnancy, i.e. decidualized endometriosis.^5^ Our case showed very scant endometrial tissue admixed with decidual cells, with the absence of the other above-mentioned features, which confirms pregnancy-related ectopic decidua of a cesarean scar.

Histopathologically, it is important to differentiate deciduosis comprising decidual cells showing varying degrees of atypia with foci of hemorrhagic necrosis from deciduoid variant of malignant mesothelioma and metastatic malignant melanoma. Ectopic decidual tissue containing myxoid stroma and vacuolated decidual cells must also be differentiated from metastatic signet ring cell carcinoma. The clinical history of the patient, the lack of active mitosis in decidual cells along with the CD10 and Progesterone Receptor (PR) positivity with non-reactivity to keratin (CK), WT1, calretinin, and HBME-1 antibody on immunohistochemistry support deciduosis.^5^^,^^7^ Table 1 depicts the differences between the above-mentioned entities.

**Table 1:** Differences between Deciduosis, Deciduoid variant of Malignant Mesothelioma, Metastatic Malignant Melanoma and Signet Cell Carcinoma

**Features**	**Deciduosis**	**Deciduoid variant of malignant mesothelioma**	**Metastatic malignant melanoma**	**Signet cell carcinoma**
Cell of origin^8^^-^11	Mesenchymal stem cells	Mesoderm	Neural crest cells	Epithelium
Most common site affected^12^^-^15	Ovaries	Peritoneum	Lung	Liver
Gender affected^2^^,^^16^^-^18	Females	Males	Males	Males
Age group most commonly affected^2^^,^^10^^,^^13^^,^^19^^,^^20^	Reproductive age (20-40 years)	5^th^ – 6^th^ decade	5^th^ decade and older	6^th^ decade
Morphology^2^^,^^11^^,^^19^^,^^21^^,^^22^	Decidualized stromal cells are polygonal, oval to spindle shaped cells with large nuclei and abundant eosinophilic cytoplasm	Malignant dyscohesive large epithelioid cells, eosinophilic cytoplasm, large round nuclei	Large epithelioid or spindle shaped, mixed cytological morphology, macro nucleoli	Signet ring cells with intracellular and extracellular mucin
Immunohistochemical features^2^^,^^11^^,^^12^^,^^21^^,^^22^	Vimentin, ER, PR, Desmin, CD 30 and CD 10 positivity	Cytokeratin (CK) MNF116, HBME-1 and Calretinin positivity	S100, HMB-45 positivity	CK20, CDX2, MUC2, MUC5AC positivity, variable MUC1 positivity
Association with occupational exposure^23^^,^^24^	Not associated	Occupational exposure to asbestos	Not associated	Not associated

ER = estrogen receptor; PR = progesterone receptor.

Deciduosis of cesarean scar also needs to be distinguished from a recently introduced entity called Deciduoma. Deciduoma is a manifestation of ectopic deciduosis; however, it is a large lesion with abundant vascularity and has a high potential for hemorrhagic complications.^1^

Additionally, our patient was a known case of hypothyroidism and GDM. The literature has shown an increased predisposition to endometriosis development in women suffering from hypothyroidism. A study conducted by Peyneau et al.^25^ showed altered metabolism of thyroid hormones in-vitro and also confirmed the aggravating role of thyroid hormones in endometriosis. Although GDM is associated with placental vasculopathy,^26^ there is no literature citing the association of GDM with deciduosis.

Very few cases of cutaneous deciduosis have been reported in literature to date. Table 2 summarizes the cases of cutaneous deciduosis reported in literature.^27^^-^35

**Table 2:** Cases of cutaneous deciduosis reported in literature to date

**Ref.**	**No of cases**	**Age**	**Site**	**AE**	**Symptoms**	**increased during pregnancy**	**IHC studies**	**Treatment**	**Follow – up**
27	01	30	CS	-	None	NR	NR	Excision on CS	NR
28	01	25	CS	-	painful nodule, 1 year previously.	Yes	Vimentin + α1antitrypsin+ Keratin -ve	Danazol until pregnancy Anti-inflammatory therapy Excision on CS	AW
29	01	40	U	+	Umbilical nodule Cyclic enlargement	-	NR	Excision on CS	Recurrence after excision
2	02	21	V	NR	Vulvar nodule, Noted during pregnancy	Yes	Vimentin +, Ki67 + PAS +	Excision	NR
		27	U	_	Umbilical nodule during current pregnancy	Yes	NR	Excision	NR
30	01	24	CS	NR	Lesion noted 2 years before	Yes	CD10 +, ER –, Calretinin +	NR	NR
31	01	36	CS	NR	Noted 2 years before	-	CK8+, hPL +, CD10+, EMA 2, PLAP 2, CK 5/6 -, calretinin -.	Excision	AW
32	02	31	Rt P	NR	Nodule noted by the patient after an uneventful vaginal delivery	NR	CD10 diffuse cytoplasmic +, Vimentin +, Pan CK and CK 8/18 -, ER weak +, PR strong +, PAS +, Colloidal Iron stain +.	Excision	NR
		26	CS	+	Tender, solid, enlarging mass in suprapubic area, superior to the cesarean incision site. Mass cyclical throbbing with the menstrual cycle.	NR	CD 10 + Vimentin + ER weak + PR strong +	Partial excision during CS, followed by excision of the remnant tissue by Panniculectomy 06 weeks later.	NR
33	01	34	CS	NR	NR	NR	NR	Excision on CS	NR
34	01	34	CS	NR	Nodule at previous cesarean scar	NR	NR	Excision on CS	NR
35	01	30	AAW	-	None	NR	NR	Excision on CS	NR
3	01	37	CS	NR	NR	NR	NR	Excision on CS	NR
This case	01	34	CS	NR	NR	NR	NR	Excision during CS	AW

AE = abdominal Endometriosis; AW = Alive and Well; CS = cesarean scar; NR = Not Reported; U = umbilicus; V = vulva; P = Perineum; AAW = Anterior Abdominal wall; CK = cytokeratin; PAS = Periodic Acid - Schiff; EMA = epithelial membrane antigen; ER = estrogen receptor; PR = progesterone receptor; PLAP = placental alkaline phosphatase; hPL = Human Placental Lactogen; MNF 116 = cytokeratin MNF116; Ki67= Labile, non-histone nuclear protein expressed in G1, S, g2 and M phase of cell cycle and then rapidly catabolized at the end of M phase, and hence, not detectable in G0 and early g1 phase cells; hence utilized as a marker of cell proliferation; Rt = right.

The mainstay of treatment of scar deciduosis is surgical excision if it fails to undergo complete remission in the post-partum period. The patients are known to completely recover following excision of the lesion, and recurrence, if present, is very rare.

Scar deciduosis is usually an uncomplicated event with an asymptomatic course. Complications, if present, are rare and may manifest in the form of rupture of the scar, with or without uterine rupture, or secondary infection of the lesion resulting in sepsis.^36^^,^^37^

## CONCLUSION

Scar deciduosis is an uncommon but possible manifestation of cutaneous endometriosis and should always be considered in an appropriate clinical setting. Although it may often mimic a neoplasm, the histopathological features of decidual cells along with the utilization of appropriate immunohistochemical techniques help to establish the diagnosis and rule out other neoplastic mimics of deciduosis.

## References

[1] Dasani M, Lee HJ, Rijhsinghani A (2019). Deciduoma, a large intrauterine mass of deciduosis. AJP Rep.

[2] Fair KP, Patterson JW, Murphy RJ, Rudd RJ (2000). Cutaneous deciduosis. J Am Acad Dermatol.

[3] Kute K, Swami S, Narwade S, Badlani K (2021). Deciduosis in a cesarean scar: a case report. Int J Clin Diagnostic Pathol..

[4] Walker A (1887). Der bau der eihaeute bei graviditatis abdominalis. Virch Arch Path Anat..

[5] Bolat F, Canpolat T, Tarim E (2012). Pregnancy-related peritoneal ectopic decidua (deciduosis): morphological and clinical evaluation. Turk Patoloji Dergisi.

[6] Zaytsev P, Taxy J (1987). Pregnancy-associated ectopic decidua. Am J Surg Pathol.

[7] Busca A, Parra-Herran C (2017). Ovary other non neoplastic ectopic decidual reaction..

[8] Valatkaitė E, Baušytė R, Vitkevičienė A, Ramašauskaitė D, Navakauskienė R (2021). Decidualization potency and epigenetic changes in human endometrial origin stem cells during propagation. Front Cell Dev Biol.

[9] Mutsaers SE (2004). The mesothelial cell. Int J Biochem Cell Biol.

[10] Turner S, Elston DM (2002). Malignant melanoma. Medscape.

[11] Gonzalez RS (2021). Colon carcinoma signet ring cell carcinoma..

[12] Kinra P, Sen A, Sharma J (2006). Ectopic decidual reaction: a case report. Med J Armed Forces India.

[13] Ordóñez NG (2012). Deciduoid mesothelioma: report of 21 cases with review of the literature. Mod Pathol.

[14] Damsky WE, Rosenbaum LE, Bosenberg M (2010). Decoding melanoma metastasis. Cancers.

[15] Mandzhieva B, Jalil A, Nadeem M, Hasan SA, Jain AG (2020). Most common pathway of metastasis of rectal signet ring cell carcinoma to the skin: hematogenous. Cureus.

[16] Karst EP, von Oiste DD (2020). Mesothelioma cancer rates in men vs women..

[17] Wyant T, Alteri R, Kalidas M, Ogoro C, Lubejko B, Eidsmoe K (2022). Key statistics for melanoma skin cancer..

[18] Wu J, Fang D, Man D (2020). Clinical correlates and prognostic value of different metastatic sites in gastric and colorectal signet ring cell carcinoma. Engineering.

[19] Santos C, Gamboa F, Fradinho F, Pêgo A, Carvalho L, Bernardo J (2012). Mesotelioma deciduóide pleural: uma entidade rara numa mulher jovem. Rev Port Pneumol.

[20] Franko J, Le VH, Tee MC (2021). Signet ring cell carcinoma of the gastrointestinal tract: national trends on treatment effects and prognostic outcomes. Cancer Treat Res Commun..

[21] Sorokin P, Nikiforchin A, Panin A, Zhukov A, Gushchin V, Kurtser M (2020). Diffuse ectopic deciduosis imitating peritoneal carcinomatosis with acute abdomen presentation: a case report and literature review. Case Rep Obstet Gynecol.

[22] Murray CA, Leong WL, McCready DR, Ghazarian DM (2004). Histopathological patterns of melanoma metastases in sentinel lymph nodes. J Clin Pathol.

[23] Fortarezza F, Della Barbera M, Pezzuto F (2021). Diagnostic challenges in epithelioid pleural mesothelioma: case series with support from electron microscopy. Diagnostics.

[24] Fritschi L, Siemiatycki J (1996). Melanoma and occupation: results of a case-control study. Occup Environ Med.

[25] Peyneau M, Kavian N, Chouzenoux S (2019). Role of thyroid dysimmunity and thyroid hormones in endometriosis. Proc Natl Acad Sci USA.

[26] Aldahmash WM, Alwasel S, Aljerian K (2022). Gestational diabetes mellitus induces placental vasculopathies. Environ Sci Pollut Res Int.

[27] Pellegrini A (1982). Cutaneous decidualized endometriosis: a pseudomalignancy. Am J Dermatopathol.

[28] Nogales FF, Martin F, Linares J, Naranjo R, Concha A (1993). Myxoid change in decidualized scar endometriosis mimicking malignancy. J Cutan Pathol.

[29] Skidmore R, Woosley J, Katz V (1996). Decidualized umbilical endometriosis. Int J Gynaecol Obstet.

[30] El-Gohary Y, Garcia M, Ganjei-Azar P (2009). Decidualized endometrioma diagnosed by fine needle aspiration cytology: a case report with immunocytochemical confirmation. Diagn Cytopathol.

[31] Val-Bernal J, Val D, Gómez-Aguado F, Corcuera M, Garijo M (2011). Hypodermal decidualized endometrioma with aberrant cytokeratin expression: a lesion mimicking malignancy. Am J Dermatopathol.

[32] Natale KE, Royer MC, Rush WL, Lupton GP (2012). Cutaneous deciduosis: a report of two cases of an unusual pseudomalignancy. J Cutan Pathol.

[33] Saeed A, Mushtaq H, Kafeel S (2013). Deciduosis in a cesarean scar. J Islam Med Dent Coll..

[34] Lingegowda J, Muddegowda P, Koteswary P, Thamilselvi R (2016). Scar endometriosis with decidual change. Natl J Basic Med Sci..

[35] Kilitçi A, Yılmaz F, Karataş A (2018). Ectopic decidua in the adipose tissue of the abdominal wall. Med Sci.

[36] Maggiore ULR, Ferrero S, Mangili G (2016). A systematic review on endometriosis during pregnancy: diagnosis, misdiagnosis, complications and outcomes. Hum Reprod Update.

[37] Sholapurkar SL, Sharp NC, Hirschowitz L (2005). Life-threatening uterine haemorrhage six weeks after Cesarean section due to uterine scar endometriosis: case report and review of literature. Aust N Z J Obstet Gynaecol..

